# Protective Effects of Voluntary Exercise on Hepatic Fat Accumulation Induced by Dietary Restriction in Zucker Fatty Rats

**DOI:** 10.3390/ijms22042014

**Published:** 2021-02-18

**Authors:** Yuka Kurosaka, Shuichi Machida, Yoko Shiroya, Hideki Yamauchi, Kumiko Minato

**Affiliations:** 1 Faculty of Health and Sports Science, Juntendo University, Chiba 270-1695, Japan; 2 Exercise Physiology Laboratory, Wayo Women’s University, Chiba 272-8533, Japan; shiro.y.109@gmail.com (Y.S.); minato@wayo.ac.jp (K.M.); 3 Department of Molecular Physiology, Division of Physical Fitness, The Jikei University School of Medicine, Tokyo 182-8570, Japan; yamauchi@jikei.ac.jp; 4 Graduate School of Health and Sports Science, Juntendo University, Chiba 270-1695, Japan; machidas@juntendo.ac.jp

**Keywords:** fatty liver, exercise, diet restriction, obesity, CD36, FABP1, hepatocyte ultrastructure

## Abstract

Weight control based on dietary restriction (DR) alone can cause lipid metabolic failure and progression to fatty liver. This study aimed to investigate the effect of exercise on preventing DR-induced hepatic fat accumulation in Zucker fatty (ZF) rats by focusing on the relationship between adipose tissue lipolysis and hepatic fat uptake. Six-week-old male ZF rats were randomly assigned to obese, DR, or DR with exercise (DR + Ex) groups. The DR and DR + Ex groups were fed a restricted diet, with the latter also undergoing voluntary exercise. After 6 weeks, hepatic fat accumulation was observed in the DR group, whereas intrahepatic fat was markedly reduced in the DR + Ex group. Compared with the obese (Ob) group, the DR group exhibited 2.09-fold expression of hepatic fatty acid translocase (FAT)/CD36 proteins (*p* < 0.01) and 0.14-fold expression of hepatic fatty acid-binding protein (FABP)1 (*p* < 0.01). There were no significant differences between the DR + Ex group and the Ob group. FAT/CD36 and hepatic triglyceride (TG) expression levels were strongly positively correlated (*r* = 0.81, *p* < 0.001), whereas there was a strong negative correlation between FABP1 and hepatic TG expression levels (*r* = −0.65, *p* < 0.001). Our results suggest that hepatic fat accumulation induced by DR in ZF rats might be prevented through exercise-induced modifications in FAT/CD36 and FABP1 expression.

## 1. Introduction

An increase in hepatic fat accumulation causes systemic metabolic abnormalities in the body, leading to the development of fatty liver, which may evolve into liver cirrhosis and a marked increase in the incidence of hepatocellular carcinoma [1]. Although nonalcoholic fatty liver tends to be a comorbidity of obesity or being overweight, hepatic fat accumulation can also develop in non-obese individuals. This tendency is especially common among Asians [2,3,4] as they have a low storage capacity for subcutaneous adipose tissue [2]. Lipids that exceed the storage capacity are speculated to accumulate in the liver and other organs and may cause fatty liver [2,5]. Furthermore, various conditions associated with severe malnutrition (e.g., kwashiorkor) can cause fatty liver disease [6]. These observations suggest that both excessive food intake and inadequate nutrition contribute to fat accumulation in the liver.

In general, both habitual exercise and dietary restriction contribute to weight loss and have an inhibitory effect on obesity and fatty liver [7,8]. It has been reported that exercise not only suppressed body weight evolution and but also improved antioxidant defense in the liver by increasing the response of oxidative metabolism markers [9]. Many studies have reported that skeletal muscle loss and fatty liver are closely related and that hepatic fat accumulation increases in sarcopenia patients and sedentary subjects [10,11,12]. We recently characterized the effects of habitual exercise and/or diet restriction on hepatic fat accumulation in Zucker fatty (ZF) rats [13,14]. ZF rats have a mutation in the leptin receptor gene that causes increased appetite and weight gain [15]. Since these rats show low physical activity levels compared with ordinary rats, they are acceptable as models for lack of exercise, and the rats eventually develop fatty liver [16,17]. Previously, we found that dietary restriction in ZF rats did not ameliorate the development of fatty liver but rather accelerated it. We also found that the same dietary restriction combined with physical exercise prevented intrahepatic fat accumulation and maintained a healthy liver [13]. Moreover, in previous studies focusing on fat cell size, it was shown that a combination of exercise and dietary restriction in ZF rats suppresses fat cell size hypertrophy and prevents fatty liver [14]. However, the mechanism through which exercise prevents dietary-restriction-induced hepatic fat accumulation is not clear.

Approximately 60% of hepatic fat is derived from free fatty acids (FFAs), which are secreted into the blood by adipocytes [18,19]. Indeed, previous studies suggested that high levels of FFA are secreted from large fat cells [20,21]. According to the findings of lipoatrophy studies [22,23] and adipose tissue restriction models [24], it has been suggested that the status of hepatic fat accumulation is related to that of adipose tissue accumulation. Serum FFA can be taken up by the liver through fatty acid translocase (FAT)/CD36, transported, and distributed by fatty acid-binding protein (FABP)1. FAT/CD36 and FABP1 are expected to play an important role in regulating hepatic fat accumulation by dietary restriction and/or exercise.

Based on these results, we hypothesized that accelerated fat accumulation in the liver in response to dietary restriction stems from impaired crosstalk between adipose tissue and the liver and that exercise could restore this. Therefore, this study aimed to investigate the effect of exercise on preventing dietary-restriction-induced hepatic fat accumulation in ZF rats, focusing on the relationship between lipolysis in adipose tissue and hepatic fat uptake.

## 2. Results

### 2.1. Animal Characteristics

**Figure 1:**
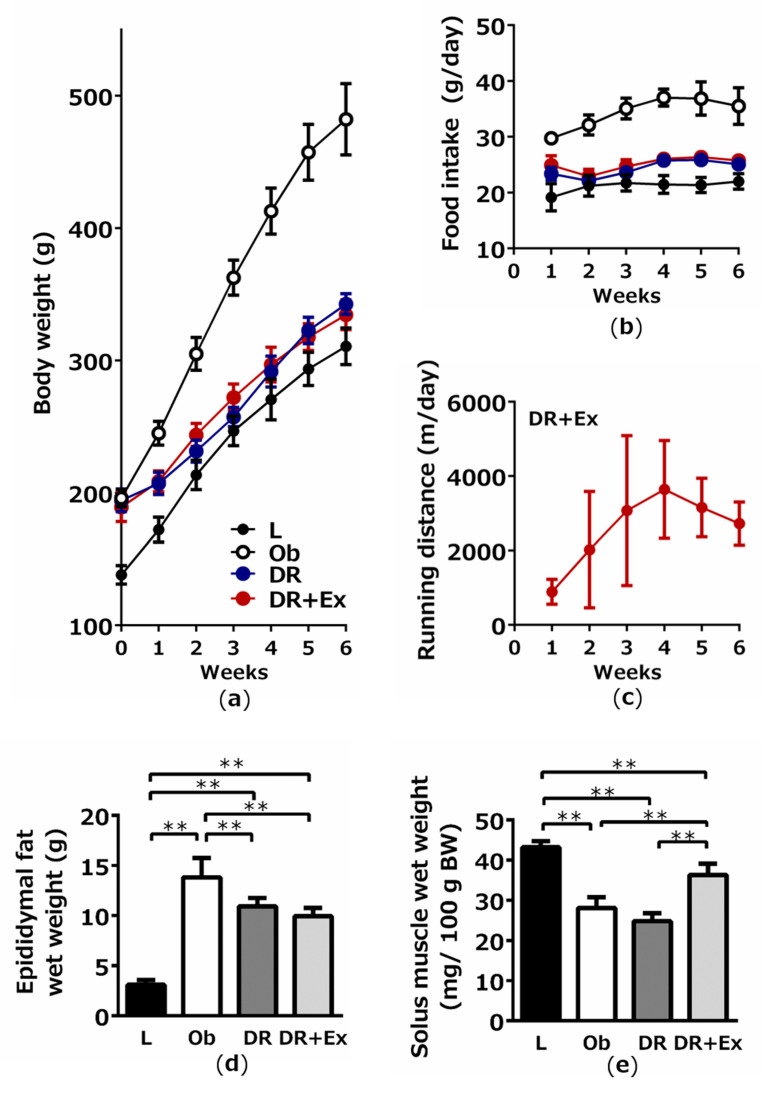
Changes in bodyweight (**a**), food intake (**b**), and running distance (**c**) during the experimental period, and final epididymal fat weight (**d**), and solus muscle weight (**e**) in lean control (L), obese (Ob), dietary restriction (DR), and dietary restriction plus exercise (DR + Ex) groups. The weight gain and fat weight were greater in the Ob group and lower in the DR and DR + Ex groups. Although rats in the DR and DR + Ex groups consumed the same amount of food, and only the DR + Ex group performed exercise (**c**), there was no significant difference in body weight between the DR and DR + Ex groups. Values are the means ± SDs (*n* = 6 rats in each group). ** *p* < 0.01.

At 12 weeks of age (end of the experiment), rats in the obese (Ob; *p* < 0.01), dietary restriction (DR; *p* < 0.05), and dietary restriction plus exercise (DR + Ex; *p* < 0.05) groups weighed significantly more than animals in the lean control (L) group (Figure 1a). Moreover, rats in the Ob group also weighed significantly more than those in the DR and DR + Ex groups (*p* < 0.01). However, body weight was not significantly different between the DR and DR + Ex groups. Mean daily food intake was significantly greater in the Ob, DR, and DR + Ex groups than in the L group (*p* < 0.01), and it was significantly lower in the DR and DR + Ex groups than in the Ob group (*p* < 0.01). However, mean daily food intake did not differ between the DR and DR + Ex groups (Figure 1b). Changes in the running distance in the DR + Ex group during the experimental period are shown in Figure 1c. The weight of the epididymal fat was significantly greater in the Ob, DR, and DR + Ex groups than in the L group (*p* < 0.01) and was significantly less in the DR and DR + Ex than in the Ob group (*p* < 0.01; Figure 1d). Moreover, the weight of the soleus muscle was significantly lower in the Ob, DR, and DR + Ex groups than in the L group (*p* < 0.01), but was significantly higher in the DR + Ex group than in the Ob and DR groups (*p* < 0.01; Figure 1e).

### 2.2. Hepatic Fat Accumulation

**Figure 2:**
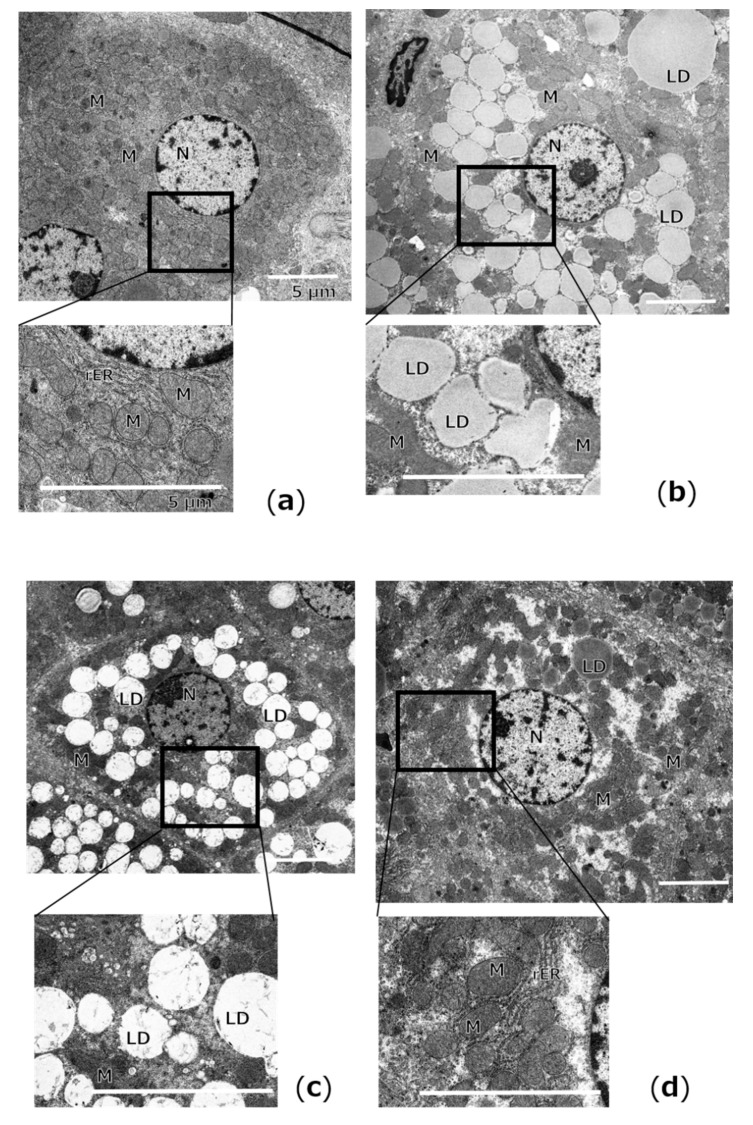
Representative images of electron micrographs of hepatocytes from the lean control (L; **a**), obese (Ob; **b**), dietary restriction (DR; **c**), and dietary restriction plus exercise (DR + Ex; **d**) groups. Lipid droplets (LD); mitochondria (M); nuclei (N); rough endoplasmic reticulum (rER). Scale bars = 5 μm.

Regarding the analysis of hepatic fat levels, representative electron micrographs of hepatocytes were examined (Figure 2). Hepatocytes in the L group showed intact organelles mostly, whereas the accumulation of numerous lipid droplets was observed within the hepatocytes of the Ob and DR groups. In contrast, lipid droplets were markedly reduced in the DR + Ex group compared with those in the Ob and DR groups. Hepatic triglyceride (TG) levels were significantly higher in the DR group than in the L (*p* < 0.01) and Ob (*p* < 0.01) groups (Figure S1a). In contrast, the hepatic TG levels in the DR + Ex group were significantly lower than those in the Ob (*p* < 0.05) and DR (*p* < 0.01) groups.

### 2.3. Serum FFA Levels

Serum FFA levels were significantly higher in the Ob and DR groups than in the L group (*p* < 0.05 and *p* < 0.01; Figure S1b). However, serum FFA levels were not significantly different between the L and DR + Ex groups.

### 2.4. Protein Expression Levels in the Epididymal Fat

**Figure 3:**
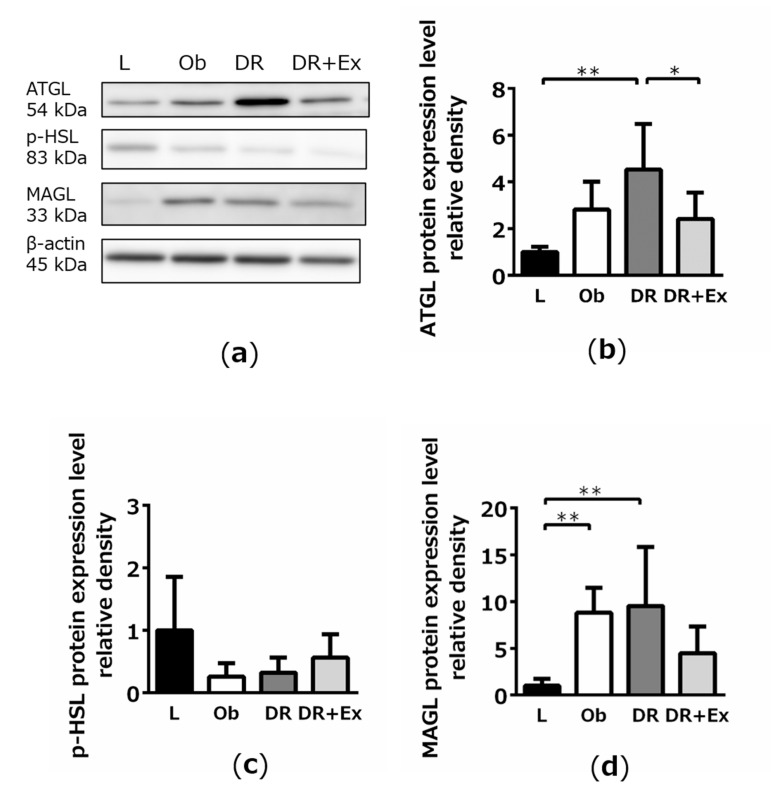
Western blotting results for the analysis of adipose triglyceride lipase (ATGL) (**b**), phosphorylated hormone-sensitive lipase (p-HSL) (**c**), and monoacylglycerol lipase (MAGL) (**d**) protein expression in lean control (L), obese (Ob), dietary restriction (DR), and dietary restriction plus exercise (DR + Ex) groups. Band images are shown in (**a**). β-Actin was used for normalization. Results are representative of three independent experiments and are shown as values relative to those in the L group, which were set to 1. Values are means ± SDs (*n* = 6 rats in each group). * *p* < 0.05, ** *p* < 0.01.

The results of Western blotting for the analysis of adipose triglyceride lipase (ATGL), phosphorylated hormone-sensitive lipase (p-HSL), and monoacylglycerol lipase (MAGL) expression in adipose tissue are shown in Figure 3a. ATGL expression levels were significantly higher in the DR group than in the L (*p* < 0.01) and DR + Ex groups (*p* < 0.05; Figure 3b), whereas p-HSL expression was not significantly different between these groups (Figure 3c). The MAGL expression levels were significantly higher in the Ob and DR groups than in the L group (both, *p* < 0.01; Figure 3d).

### 2.5. Protein Expression Levels in the Liver

**Figure 4:**
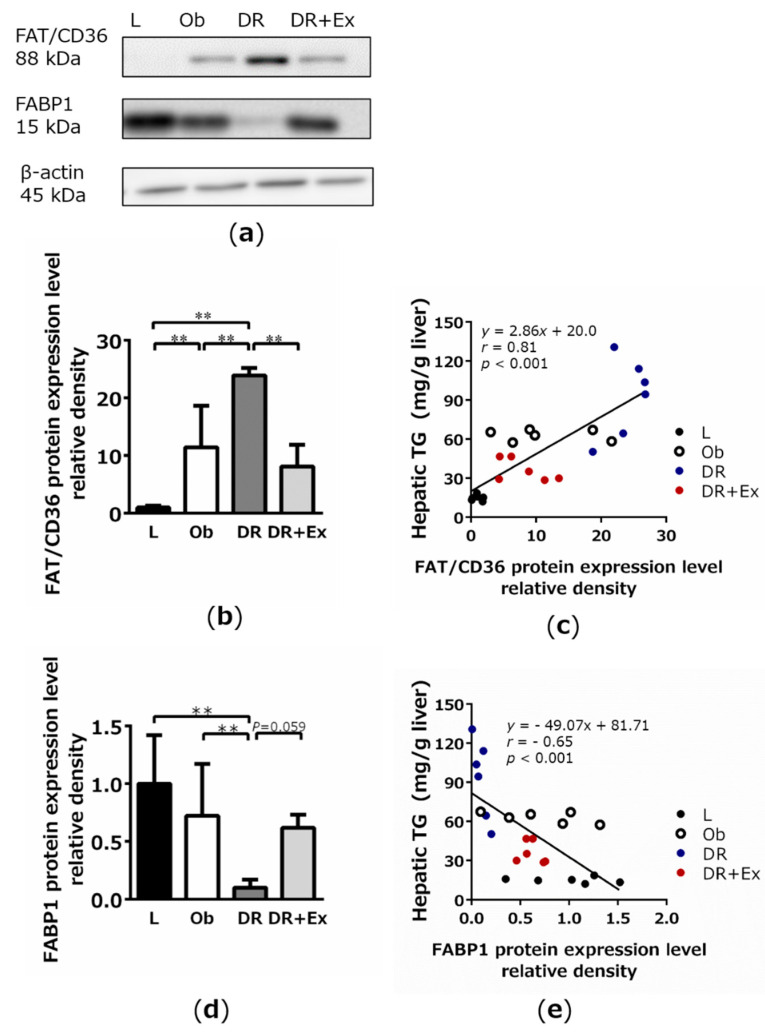
Results of Western blotting for the analysis of hepatic fatty acid translocase (FAT/CD36) (**b**) and fatty acid-binding protein (FABP)1 (**d**) protein expression in lean control (L), obese (Ob), dietary restriction (DR), and dietary restriction plus exercise (DR + Ex) groups. Band images are shown in (**a**). The correlation between hepatic triglycerides (TGs) and the expression of each protein is shown (**c**) and (**e**). β-Actin was used for normalization. Each protein expression result represents three independent experiments, and these values are shown as values relative to those in the L group, which were set to 1. Values are means ± SDs (*n* = 6 rats in each group). ** *p* < 0.01.

The Western blotting results for the analysis of hepatic fatty acid translocase (FAT)/CD36 and FABP1 protein expression are shown in Figure 4a. The FAT/CD36 expression levels were significantly higher in the Ob and DR groups than in the L group (*p* < 0.01) and significantly higher in the DR group than in the Ob (*p* < 0.01) and DR + Ex groups (*p* < 0.01; Figure 4b). In addition, a strong correlation was noted between FAT/CD36 expression levels and hepatic TG levels (r = 0.81, *p* < 0.01; Figure 4c). The FABP1 expression levels were significantly lower in the DR group than in the Ob group (*p* < 0.01; Figure 4d). The FABP1 expression levels were higher in the DR + Ex group than in the DR group, though the difference was not statistically significant (*p* = 0.059; Figure 4d). Moreover, a strong negative correlation was noted between FABP1 expression levels and hepatic TG levels (r = −0.63, *p* < 0.01; Figure 4e).

## 3. Discussion

We examined the effect of voluntary exercise on the prevention of hepatic fat accumulation by dietary restrictions in ZF rats. The characteristic of this experimental design was that body weight was controlled similarly among groups. Dietary restrictions in ZF rats improved blood TG and insulin resistance but have already been reported to exacerbate fatty liver [13,14]. This result is different from that of other rat strains [7,8,25] and may be a phenomenon specific to this particular rat strain. Other characteristics of ZF rats include low physical activity [17] and skeletal muscle developmental disorders [26]; therefore, the ZF rat might be useful as a fatty liver model in sedentary people. In the present study, dietary restriction led to accelerated fatty liver development via high expression of ATGL in the adipose tissue and FAT/CD36 in the liver and low expression of FABP1 in the liver. Moreover, exercise was found to suppress the expression of the dietary-restriction-induced molecules mentioned above, as well as hepatic fat accumulation.

FAT/CD36 and FABP1 are the main transporters for FFA in the liver. FAT/CD36 is a transmembrane protein that accelerates FFA uptake via facilitated diffusion [27,28]. Although this protein is generally not highly expressed in the liver, it is enhanced by diet-induced obesity. In previous studies, the histological findings of the relationships between nonalcoholic steatohepatitis and blood FFA and liver FAT/CD36 levels were reported based on analyses of tissues extracted from liver biopsies of obese people [29,30]. Reports on the elevated expression of FAT/CD36 in high-fat, diet-induced fatty liver also exist [31]. In addition, it was reported that FAT/CD36-deficient mice show resistance to fatty-liver-inducing dietary factors, such as high carbohydrate intake and alcohol load [32]. Hence, it is understood that FAT/CD36 plays an important role in the regulation of hepatic fat accumulation. In this study, FAT/CD36 was overexpressed in obese ZF rats and further increased by dietary restriction.

Moreover, FFA incorporated into hepatocytes via FAT/CD36 is transported and distributed by FABP1 [33,34]. However, it has been reported that FABP1 increases [35] or does not change [36] during simple hepatic fat accumulation. Numerous reports note that FABP1 is overexpressed in the early stage of liver disease and downregulated in advanced liver disease [30]. The suppression of FABP1 has been shown to potentially exacerbate lipotoxicity and liver disease progression [33]. In this study, no significant differences in FABP1 levels were observed between the L, Ob, and DR + Ex groups. The increase in FABP1 in the DR group constitutes one of the adverse effects of dietary restriction under low activity conditions. In this study, protein expression of FABP1 was significantly suppressed by dietary restriction, whereas fat accumulation in the liver progressed the most in the DR group of rats. By dietary restriction, FABP1 was suppressed, and the capacity for FFA transport and distribution regulation was considered inferior. This led to metabolic abnormalities, suggesting that FFA was stagnant in hepatocytes. The consistently low FABP1 expression by dietary restriction, and substantially high hepatic TG compared with those in the other groups, strongly supports the role of FABP1 in the development of fatty liver. This is consistent with a report by Charlton M et al. [30], which revealed that FABP1 levels are also reduced in advanced nonalcoholic steatohepatitis and may cause lipotoxicity in hepatic fat accumulation.

In fact, from ultrastructural observation, numerous lipid droplets of various sizes were observed in the hepatocytes of obese ZF rats. This trend was further intensified by dietary restriction, which correlates with the results of hepatic TG levels. Furthermore, morphological abnormalities of cell organelles were observed, suggesting the possibility of their dysfunction. In this study, there was a strong positive correlation between hepatic TG and FAT/CD36, and, conversely, a strong negative correlation between hepatic TGs and FABP1. FAT/CD36 and FABP1 are related to the deterioration of fatty liver by dietary restriction. In addition, rats with suppressed hepatic fat accumulation by the same dietary restriction combined with physical exercise had the opposite results. These results suggest that exercise suppresses dietary-restriction-induced hepatic fat accumulation.

The increase in serum FFA by dietary restriction, identified in this study, was presumably the result of enhanced lipolysis in adipose tissues. The increased expression of the proteins ATGL, p-HSL, and MAGL in adipose tissues is known to cause an increase in lipolysis rate and activity [29,30]. The expression of ATGL and MAGL was high under dietary restriction. This sequence of events that starts with dietary restriction and results in adipose tissue lipolysis may have caused a concomitant increase in serum fatty acid levels. This increase may have led to the influx and accumulation of fatty acids in the liver instead of their metabolization (to obtain energy) or redirection to other organs, where they serve as an important source for TG synthesis. When dietary restriction was combined with exercise, this sequence of events was found to be suppressed.

Although dietary restrictions work effectively under high physical activity conditions [7,8,25], it seems dietary restrictions under conditions of low physical activity, like that in this study, may cause hepatic fat accumulation-related metabolic disorders. Some hepatic fat metabolic disorders that arise due to lack of exercise have been reported [37], along with many positive effects of exercise on hepatic metabolism [38]. In this study, the effects of dietary restriction on fatty liver formation were confirmed, which may largely be due to low physical activity and poor skeletal muscle development in ZF rats. Although the running distance of the DR + Ex group was short, their skeletal muscle mass relative to body weight was closer to that of the L group, and hepatic fat accumulation was suppressed. This shows the effect the right amount of activity may have on reversing hepatic fat accumulation caused by dietary restriction.

There are some limitations to this study. First, the study used ZF rats, in which overeating is induced by mutations in the leptin receptor [39,40]. Further, the protective effects clarified in this study have not been confirmed in other fatty liver models, and the possibility of applications to humans is an issue for the future. It will be necessary to examine the effects of dietary restrictions on skeletal muscle developmental disorders in low physical activity models other than ZF rats. Second, there was no exercise-only group. This is because our preliminary studies showed that exercise alone could not induce the same level of suppression with respect to body weight gain compared to dietary restriction. Finally, while the data presented here suggest an association from static measurements, we believe that the mechanistic details of the protective effects of voluntary exercise on DR-induced hepatic fat accumulation must be elucidated. In future studies, based on the results of this work, it would be necessary to assess fatty acid uptake and lipolysis in several experimental models.

Of note, in the DR and DR + Ex groups in this study, weight gain was suppressed to the same extent by different weight control means. This study suggests that the means used to control body weight (dietary-restriction-only or in combination with exercise) are important and have implications with respect to protein expression related to adipose lipolysis and hepatic fatty acid transport. It is clear that weight control by dietary restriction alone might increase the risk of steatosis through adipose tissue lipolysis and fatty acid transport to the liver in ZF rats, indicating that dietary restriction combined with exercise is necessary for protection from DR-induced hepatic fat accumulation.

In conclusion, our results suggest that dietary-restriction-induced hepatic fat accumulation in ZF rats might occur via the modulation of FAT/CD36 and FABP1. Further, exercise may have protective effects against hepatic fat accumulation induced by dietary restriction in Zucker fatty rats.

## 4. Materials and Methods

### 4.1. Experimental Design

The experimental procedure was reported by Kurosaka et al. [14]. Our study was approved by the Biological and Epidemiologic Research Committee for Animal Use of Wayo Women’s University (No. 1015, Ichikawa, Japan, 2 February 2011). All procedures and protocols followed the standard guidelines for the care and use of laboratory animals.

Male Zucker lean rats and male ZF rats were purchased at 5 weeks of age from Charles River Laboratories Japan, Inc. (Yokohama, Japan). All animals were housed in individual cages in a temperature-controlled (21.8 ± 0.6 °C) animal room with a 12-h light (08:00–20:00)/dark (20:00–08:00) cycle. Following a 1-week acclimation period, 18 ZF rats, aged 6 weeks (174.3–204.5 g weight), were allocated to each of the three groups (Ob, DR, or DR + Ex groups) using computer-generated randomization. Moreover, six Zucker lean rats, aged 6 weeks (non-hyperphagic strain, 132.0–148.2 g weight), comprised the lean control (L) group. All animals received standard rodent chow (NMF; Oriental Yeast Co., Ltd., Tokyo, Japan) based on the allowed quantity of food for each animal and were allowed ad libitum access to water. Body weight and food consumption were monitored daily throughout the study period. The animals in the DR group received 70% of the mean volume of food consumed by the animals in the Ob group. The animals in the DR + Ex group were subjected to dietary restriction and were allowed unrestricted access to a running wheel. The running exercise was voluntary, and the running distance was measured with a running-wheel exercise device associated with an ergometer, equipped within the housing space (Yamashita Giken, Tokushima, Japan). Furthermore, the quantity of food was regulated to allow equivalent weight gain in the DR + Ex and DR groups. The L and Ob groups were maintained under sedentary conditions and had free access to food.

In this study, additional experiments were conducted using samples obtained from previous research (*n* = 2 rats in each group) [14] and newly added samples (*n* = 4 rats in each group).

### 4.2. Sampling

At 12 weeks of age, all animals were fasted for 12 h to exclude the influence of diet up until the day before, and the running wheels of the DR + Ex group were locked. The rats were anesthetized with isoflurane, and blood samples were collected into serum gel separator tubes (VENO-JECT II plastic vacuum tube; Terumo, Tokyo, Japan) and centrifuged to separate the serum for blood chemistry tests. The liver and epididymal adipose tissues were resected from the anesthetized rats. Samples of adipose tissue and hepatic tissue were frozen in liquid nitrogen and stored at −80 °C until analysis. The rats were then sacrificed by fatal exsanguination.

### 4.3. Serum Analysis

Serum levels of FFAs were measured using commercially available kits according to the manufacturer’s instructions (NEFA E-test, Wako Pure Chemical Industries Ltd., Osaka, Japan).

### 4.4. Liver Tissue Preparation for Triglyceride (TG) Analysis

To extract lipids, frozen liver samples were powdered and homogenized with a 2:1 (*v/v*) chloroform–methanol mixture [41]. Hepatic TG levels were determined using a kit (TG E-Test; Wako Pure Chemical Industries Ltd., Osaka, Japan).

### 4.5. Electron Microscopy

Hepatic tissues were fixed in 2.5% glutaraldehyde in 0.1 M cacodylate buffer, pH 7.4, post-fixed in 1% OsO4 in the same buffer, dehydrated in a graded series of ethanol, and embedded in Epon 812. Ultrathin sections were cut, stained with uranyl acetate followed by lead citrate, and examined with a JEOL 1220 electron microscope (JEOL Ltd., Tokyo, Japan) operating at 80 kV [42].

### 4.6. Western Blotting

The expression of FAT/CD36 and FABP1 in the liver and the expression of ATGL, p-HSL, and MAGL in the adipose tissue were examined by Western blotting. Frozen hepatic tissues (approximately 60–70 mg) were homogenized in seven volumes, and frozen epididymal fat tissues (approximately 100–110 mg) were homogenized in four volumes of ice-cold homogenization buffer (Chaps buffer; Cell Signaling Technology Inc. (CST), Danvers, MA, USA) containing phenylmethylsulfonylfluoride (CST), dithiothreitol (Wako Pure Chemical Industries Ltd., Osaka, Japan), protease inhibitor (Roche Diagnostics GmbH, Mannheim, Germany), and phosphatase inhibitor (Roche Diagnostics GmbH). Homogenates were centrifuged at 10,000× *g* for 20 min at 4 °C to collect supernatants. Protein concentrations were determined using a BCA Protein Assay Kit (Bio-Rad Laboratories, Irvine, CA, USA). Approximately 10 μg of total protein was applied to each lane on 7.5–15% polyacrylamide gels and separated by sodium dodecyl sulfate–polyacrylamide gel electrophoresis (SDS-PAGE). Proteins were then electrotransferred onto polyvinylidene difluoride membranes. Membranes were blocked with 5% nonfat dry milk and then incubated for 1 h at room temperature with the following primary antibodies: anti-FAT/CD36 (1:1000; Santa Cruz Biotechnology), anti- FABP1 (1:1000; CST), anti-ATGL (1:1000; Abcam), anti-p-HSL (1:1000; CST), anti-MAGL 1:200; Abcam), and anti-β-actin (1:1000; CST). Membranes were then incubated with anti-rabbit horseradish peroxidase-conjugated secondary antibodies (1:4000; CST) for 1 h at room temperature. Proteins were detected using ECL Prime reagent (GE Healthcare, Piscataway, NJ, USA). The signals were captured using an imaging system (GE Healthcare). Densitometry analysis was conducted using Image Quant TL (Ver. 8.1) software (GE Healthcare).

### 4.7. Statistical Methods

All statistical analysis was performed using IBM SPSS (Statistical Package for the Social Sciences) statistics 26 (SPSS Inc., Chicago, IL, USA). All results were confirmed to be normally distributed using the Kolmogorov–Smirnov test. A one-way analysis of variance was performed for each outcome measure. Significant main effects were followed up using Bonferroni post hoc comparisons. Pearson’s test was adopted to examine the correlation between the protein expression level and liver TG levels. All values are expressed as means ± standard deviation (SD); *p* < 0.05 indicates statistical significance.

## Supplementary Materials

The following are available online at https://www.mdpi.com/1422-0067/22/4/2014/s1, Figure S1: Hepatic triglyceride levels and serum free fatty acid.
