# Supplementary material for: Protective Effects of Voluntary Exercise on Hepatic Fat Accumulation Induced by Dietary Restriction in Zucker Fatty Rats
Source: Int J Mol Sci. 2021 Feb 18;22(4):2014. doi: 10.3390/ijms22042014 (PMC7922922; doi:10.3390/ijms22042014)
Supplement: Supplementary file 1 [file ijms-22-02014-s001.pdf]

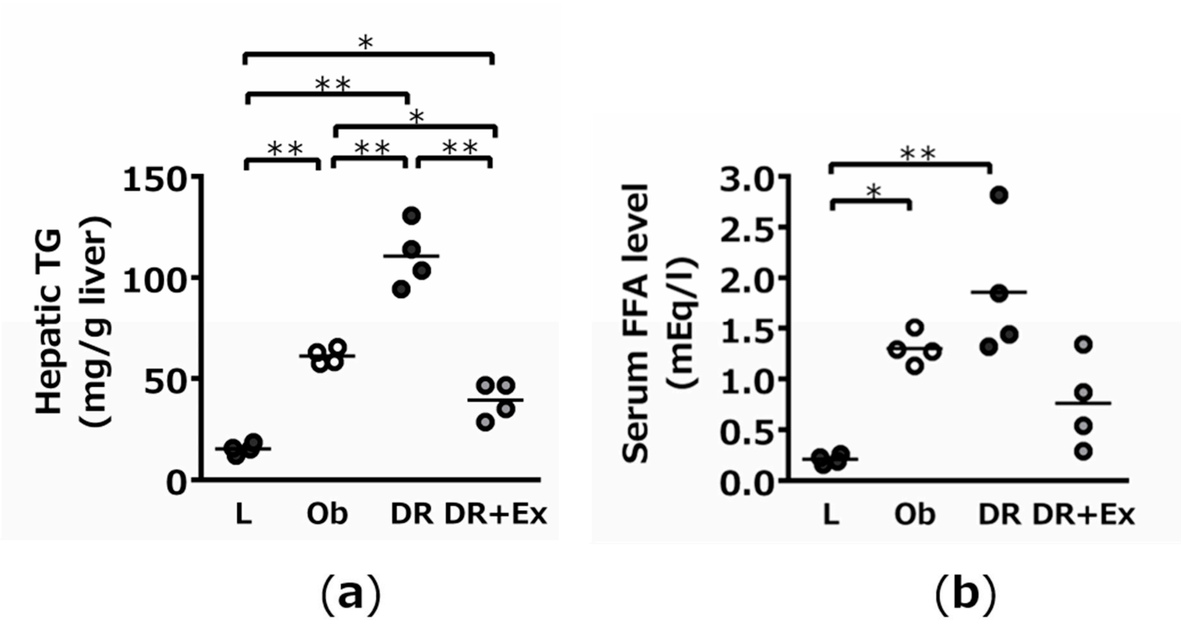

Figure S1: Hepatic triglyceride (TG) levels (**a**) and serum free fatty acid (FFA) levels (**b**) in lean control (L), obese (Ob), dietary restriction (DR), and dietary restriction + exercise (DR+Ex) groups. Since two animals had already been reported in a previous study, the statistical analysis was performed on the unreported data of four animals. Kruskal-Wallis test of variance was performed for each outcome measure. Significant main effects were followed up using Bonferroni post hoc comparisons. The same tendency as that observed in a previous study [14] was confirmed. Data shown by each data plot and mean. (n = 4 rats in each group). *$P < 0.05$, **$P < 0.01$.
